# Potentials of Acetylcholinesterase and Butyrylcholinesterase Alterations in On-Pump Coronary Artery Bypass Surgery in Postoperative Delirium: An Observational Trial

**DOI:** 10.3390/jcm12165245

**Published:** 2023-08-11

**Authors:** Thomas S. Zajonz, Christian Kunzemann, Anna Lena Schreiner, Frauke Beckert, Emmanuel Schneck, Andreas Boening, Melanie Markmann, Michael Sander, Christian Koch

**Affiliations:** 1 Department of Anesthesiology, Operative Intensive Care Medicine and Pain Therapy, University Hospital of Giessen and Marburg, Justus Liebig University of Giessen, 35392 Giessen, Germany; christian.kunzemann@chiru.med.uni-giessen.de (C.K.); anna-lena.c.schreiner@chiru.med.uni-giessen.de (A.L.S.); frauke.beckert@chiru.med.uni-giessen.de (F.B.); emmanuel.schneck@chiru.med.uni-giessen.de (E.S.); melanie.markmann@chiru.med.uni-giessen.de (M.M.); michael.sander@chiru.med.uni-giessen.de (M.S.); christian.koch@chiru.med.uni-giessen.de (C.K.); 2 Department of Cardiac and Vascular Surgery, University Hospital of Giessen and Marburg, Justus Liebig University of Giessen, 35392 Giessen, Germany; andreas.boening@chiru.med.uni-giessen.de

**Keywords:** intensive care, postoperative delirium, cardiac surgery, preoperative patient evaluation and improvement, cardiopulmonary bypass, postoperative care

## Abstract

Cardiac surgery is regularly associated with postoperative delirium (POD), affected by neuro-inflammation and changes in cholinergic activity. Therefore, this prospective observational study aimed to evaluate whether pre- and perioperative changes in blood acetylcholinesterase (AChE) and butyrylcholinesterase (BChE) activity were associated with POD development in patients undergoing isolated elective coronary artery bypass graft (CABG) surgery. It included 93 patients. Pre- and postoperative blood AChE and BChE activities were measured with photometric rapid-point-of-care-testing. The Intensive Care Delirium Screening Checklist and the Confusion Assessment Method for the Intensive Care Unit were used to screen patients for POD. POD developed in 20 patients (21.5%), who were older (*p =* 0.003), had higher EuroSCOREs (*p* ≤ 0.001), and had longer intensive care unit stays (*p* < 0.001). On postoperative day one, BChE activity decreased from preoperative values more in patients with (31.9%) than without (23.7%) POD (group difference *p* = 0.002). Applying a cutoff of ≥32.0% for BChE activity changes, receiver operating characteristic analysis demonstrated a moderate prediction capability for POD (area under the curve = 0.72, *p* = 0.002). The risk of developing POD was 4.31 times higher with a BChE activity change of ≥32.0% (*p* = 0.010). Monitoring the pre- to postoperative reduction in BChE activity might be a clinically practicable biomarker for detecting patients at risk of developing POD after CABG surgery.

## 1. Introduction

Delirium represents an acute and fluctuating disturbance of cerebral function associated with changes in consciousness and attention. It may be classified according to various features, such as predominant motor activity (hypoactive, hyperactive, or mixed) or etiology (hypoxia, sepsis, sedative exposure, or metabolic dysfunction) [1]. It increases morbidity and mortality, considerably impacting public health costs [2,3].

Postoperative delirium (POD) is common in cardiac surgery: its incidence approaches 40%, increasing with age [2,4].

The POD diagnosis is still confirmed by clinical tests without predictive potential [5,6]. POD development after cardiac surgery is influenced by patient factors, including age, mental health, and neurological status, and procedural factors, such as surgical trauma, extracorporeal circulation duration, and anesthetic management, resulting in neurotransmitter dysregulation and cerebral network dysconnectivity [2,4,7,8,9,10].

The neuronal and non-neuronal cholinergic system is pivotal in neurological and immunological signal transduction in the bidirectional communication between central and peripheral neuronal circuits, immune cells and cytokines, neuro-endocrine hormonal systems, and gut microbiota and their metabolites [11]. The high density of cholinergic synapses in the thalamus, striatum, limbic system, and neocortex suggests that cholinergic transmission is likely critically important for consciousness, memory, learning, attention, and other higher brain functions [12]. Based on the bi-directional cross-talk between the central nervous system and peripheral neuronal circuits, cholinergic homeostasis can be disturbed by immunologic and inflammatory stressors in the context of surgical interventions.

Changes in the cholinergic system, its activity, and POD development have been demonstrated in various settings, including cardiac surgery [13,14,15,16,17,18]. However, those results are inconsistent regarding the importance of the two primary investigated CHE subtypes—acetylcholinesterase (AChE) and butyrylcholinesterase (BChE)—and the influence of preoperative and perioperative activity patterns on POD development.

For example, John et al. [13] found no difference in AChE and BChE activity in cardiosurgical patients with or without POD, while Adam et al. [17] promoted the theory that the cholinergic deficit hypothesis may be of importance for POD development. However, it should be noted that both studies examined non-uniform surgical intervention groups.

This study aimed to examine whether pre- and perioperative changes in blood AChE and BChE activity were associated with POD in a patient group with a uniform surgical intervention. We examined a prolonged analysis period until the seventh postoperative day. Furthermore, we aimed to identify potential cofactors affecting POD after isolated CABG surgery.

## 2. Materials and Methods

This observational trial included 100 patients at the University Hospital of Giessen and was registered in the German Clinical Trials Register (trial registration: DRKS00010959). Local ethics committee approval was obtained (Justus-Liebig-University Giessen, Giessen, Germany; approval number AZ: 30/16). Written informed consent was obtained from all patients. The study was conducted according to the principles of the Declaration of Helsinki [19]. The methods and results are presented according to the Strengthening the Reporting of Observational Studies in Epidemiology (STROBE) guidelines [20].

### 2.1. Subjects

Patients were enrolled between September 2016 and January 2020. All patients underwent elective CABG surgery and postoperative care in the intensive care unit (ICU). Inclusion criteria were age ≥18 years, elective on-pump CABG surgery, and the ability to communicate in German or English. Exclusion criteria included: missing consent, denial of participation, pregnancy, preoperative atrial fibrillation, severe bradycardia (<60bpm; types: sinus bradycardia, atrial fibrillation with low frequency, nodal rhythm, and second- or third-degree atrioventricular block), acute infection before surgery, pre-existing autoimmune disease, immunomodulatory medication, left ventricular ejection fraction <30%, and renal insufficiency (Kidney Disease Improving Global Outcome score >2). We also excluded patients with pseudocholinesterase deficiency, preoperative delirium (screened using the Confusion Assessment Method for the Intensive Care Unit [CAM-ICU] and/or Intensive Care Delirium Screening Checklist [ICDSC]), cognitive dysfunction (e.g., history of schizophrenia, other severe psychiatric conditions, or dementia with inability to answer the CAM-ICU or ICDSC in an adequate manner), and recent or persistent neurologic impairment (e.g., acute cerebral infarction, intracranial bleeding, or acute meningitis in the last three months prior to study inclusion leading to an inability to answer the CAM-ICU or ICDSC).

### 2.2. Preoperative Assessment

Each patient was initially examined the evening before surgery. A questionnaire-based history was obtained to survey somatic (age, sex, weight, and body mass index) and medical (comorbidities including atrial fibrillation, chronic obstructive pulmonary disease [COPD], and diabetes mellitus type II; American Society of Anesthesiologists [ASA] physical status; New York Heart Association classification; and smoking habits, alcohol consumption, drug abuse, and medication addiction) factors. The Euroscore was calculated with the help of the EuroSCORE calculator provided by the Royal Papworth Hospital [21]. The initial examination comprised screening for delirium using the ICDSC and CAM-ICU.

### 2.3. Quantification of AChE and BChE

A preoperative baseline analysis of AChE and BChE activities was performed immediately after the clinical examination and peripheral intravenous line placement, as well as the consecutive seven postoperative days. Whole blood samples (10 µL) were drawn from this peripheral intravenous catheter and analyzed using the ChE Check Mobile System (Securetec Detektions-Systeme AG, Neubiberg, Germany). Analysis of AChE and BChE activity was performed by following the procedural steps according to manufacturer instructions (for detailed description, see [22,23]). The other laboratory data were derived from clinical routine laboratory blood sampling.

### 2.4. Intraoperative Assessment

The following data were recorded: duration of anesthesia, ventilation, surgery, cardiopulmonary bypass (CPB), and aortic cross-clamping; anesthesia method; drugs used; and transfusions.

### 2.5. Postoperative Assessment

All patients were transferred to the ICU under mechanical ventilation. Then, a resident with experience using the applied delirium screening tests and the CHE activity analyzer performed the following examinations. After extubation, POD development was monitored for seven days using the ICDSC and CAM-ICU. A patient with a positive result with either instrument was considered to have POD. Testing for POD was performed on postoperative days 1–7. An investigator performed all examinations to rule out bias under the supervision of the principal investigator and the attending intensive care physicians. They based their assessment on the ICD-10 criteria [24]. Sedation status was examined using the Richmond Agitation-Sedation Scale: patients with a score ≤−2 were excluded from testing and reevaluated after four hours. After POD testing was completed, AChE and BChE activities were analyzed identically to preoperative sampling. Postoperative pain was routinely measured using the Visual Analog Scale at every observation point. Other recorded data included duration of mechanical ventilation, length of ICU and hospital stay, in-hospital mortality, and blood transfusion; the other laboratory data were derived from clinical routine laboratory blood sampling (alkaline phosphatase [ALP, range 40–130 U/L], alanine transaminase [ALT, normal range 10−50 U/L], C-reactive protein [CRP, normal range <0.5 mg/L], gamma-glutamyltransferase [GGT, 10−66 U/L], hemoglobin A1c [HBA1c, normal range <5.6%], glucose [normal range 60−110 mg/dL], hemoglobin [normal range 13.5−17.2 g/dL], thrombocytes [normal range 150−370 G/L], erythrocytes [normal range 4.3−5.8 G/L], hematocrit [normal range 0.39−0.51%], and mean corpuscular hemoglobin concentration [normal range 27−33.5 G/L]).

### 2.6. Statistical Analysis

Data were entered into an Excel database (Microsoft, Redmond, WA, USA) and mean, standard deviation, median, and IQR were subsequently calculated for POD+/POD−. Data distribution was evaluated with the Shapiro–Wilk’s test. Differences between groups were compared by Student’s *t*-test or Wilcox test according to normal distribution for numerical data and by Fisher’s exact test for categorical data. Postoperative BChE and AChE activity measurements are expressed as percentages relative to the preoperative values (set as 100%). Differences in absolute BChE and AChE activities were compared between groups using analyses of variance with post hoc Tukey’s honestly significant difference tests. The true positive (TPR; sensitivity) and false positive (FPR = 1–TPR; specificity) rates with different cutoffs for the decrease in BChE activity were plotted as a receiver operating characteristic (ROC) curve, where each point represents a sensitivity/specificity pair corresponding to a particular cutoff. The threshold value was determined by optimal Youden index. ROC analysis was performed to calculate diagnostic accuracy using the ROCit package (version 2.1.1, R Core Team, https://www.r-project.org/, accessed on 16 June 2023). R: A language and environment for statistical computing. R Foundation for Statistical Computing, Vienna, Austria) in R. A *p* < 0.05 was considered statistically significant. Logistic regression analysis was used to infer the significance of influence of BCHE and ACHE levels with occurrence of POD and, subsequently, Fisher test was used to examine the different distribution of BCHE values that dropped below certain thresholds among the POD− and POD+ group. ORs were retrieved from Fisher Test analysis. All analyses were performed using R Statistical Software (version 4.0.4; R Core Team 21, https://www.r-project.org/, accessed on 16 June 2023).

## 3. Results

### 3.1. Patient Sample

After assessing 100 patients for study eligibility, 3 were excluded because of pre-existing delirium, and 4 declined to participate. Therefore, 93 patients were included for analysis.

### 3.2. Baseline Characteristics

All patients received balanced anesthesia with propofol/etomidate, pancuronium, sufentanil, and sevoflurane/isoflurane. Most participants were male (87.1%). Eight patients (8.6%) had a history of cerebral infarction without persistent neurological impairment. Five patients had a history of psychiatric disorder (5.4% depression, post-traumatic stress disorder, or obsessive–compulsive disorder). No drug or medication abuse was reported. Thirty-one patients (33.3%) reported regular alcohol consumption. ASA physical status classification was III or IV in 92.5% of patients.

### 3.3. Incidence of Delirium

**Figure 1:**
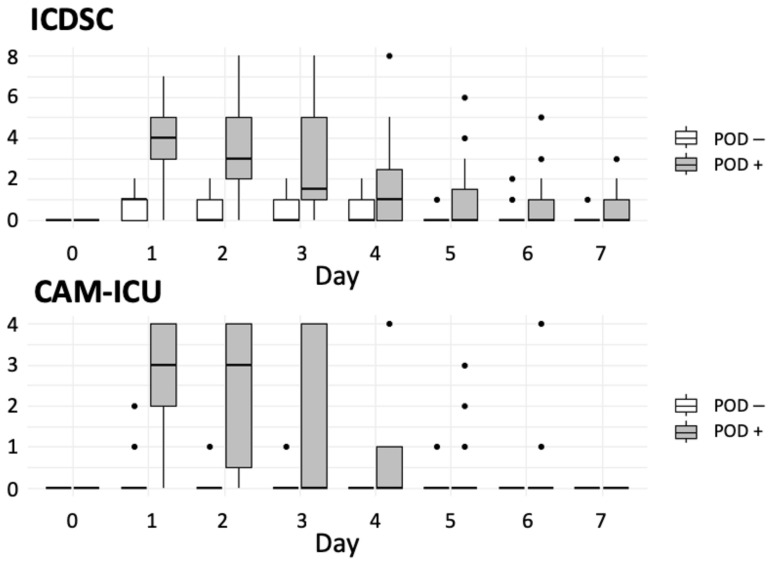
Chart demonstrating the recorded points of the used screening tools at the study time points. Abbreviations: CAM-ICU, confusion assessment method–intensive care unit; ICDSC, intensive care delirium screening checklist; POD, postoperative delirium.

POD developed in 20 patients (21.5%). CAM-ICU and ICDSC results were consistent in 94.6% of patients. In five patients (5.4%), CAM-ICU differed from the ICDSC results. In these cases, the attending intensivist diagnosed or ruled out POD based on ICD-10 classification. POD developed primarily on the first postoperative day (Figure 1).

### 3.4. Intergroup Differences

#### 3.4.1. Preoperative Variables

Patients who developed POD (POD+) were significantly older (*p* = 0.003) and had a higher EuroSCORE (*p* < 0.001) than those without POD (POD−). The proportion of patients with type II diabetes mellitus was significantly higher in the POD+ than in the POD− group (*p* = 0.008). HbA1c (*p* = 0.003) and blood glucose (*p* = 0.039) levels were significantly higher in the POD+ than in the POD− group. Group-wise patient characteristics are shown in Table 1. Other blood parameters did not differ significantly in the POD+ and POD− group (Table 2).

#### 3.4.2. Intraoperative Variables

Intraoperative hemoglobin (*p* =0.016), thrombocyte count (*p* = 0.042), and GGT level (*p* = 0.018) were significantly lower in the POD+ than in the POD− group (Table 2). CPB (*p* < 0.001), aortic cross-clamping (*p* = 0.011), and intraoperative ventilation (*p* < 0.001) durations were significantly longer in the POD+ than in the POD− group. The anesthesia and surgery durations did not differ significantly between the groups (Table 3).

#### 3.4.3. Postoperative Variables

Erythrocyte count (*p* = 0.009), hemoglobin concentration (*p* = 0.002), mean corpuscular hemoglobin concentration (*p* =0.002), thrombocyte count (*p* =0.015), and hematocrit (*p* = 0.016) were significantly lower in the POD+ than in the POD− group. The red blood cell transfusion volume was significantly higher in the POD+ than in the POD− group (*p* < 0.001).

ALP (*p* = 0.009) levels were significantly lower in the POD+ than in the POD− group, whereas GGT levels did not differ significantly (p = 0.076). Ventilation duration (*p* < 0.001) and ICU stay length (*p* < 0.001) were significantly longer in the POD+ than in the POD− group (Table 3). The CRP level and leucocyte count did not differ significantly between groups.

#### 3.4.4. AchE and BChE Activity

Preoperative BChE and AChE activities did not differ significantly between the POD+ (BChE = 3158.5 [3018.3–3512.1] U/g Hb; AChE = 42.6 [40.3–47.4] U/g Hb) and POD− (BChE = 3180.9 (2822.9–3472.1) U/g Hb; AChE = 45.1 [42.3–48.3] U/g Hb) groups.

**Figure 2:**
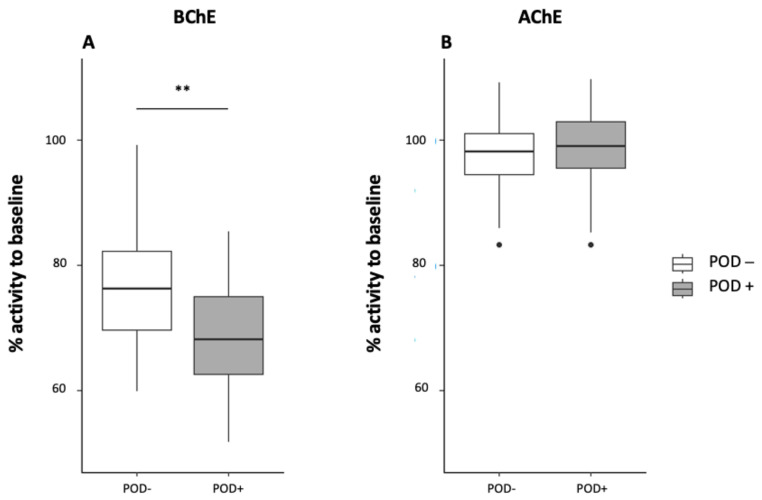
Butyrylcholinesterase (BChE) (**A**) and acetylcholinesterase (AChE) activity (**B**) on postoperative day one shown as a percentage of baseline activity measured before surgery. Key: **, *p* = 0.002.

BChE activity decreased significantly from preoperative values in both groups on postoperative day one. BChE activity decreased significantly to 2,189.3 (1917.5–2364.9) U/g Hb in the POD+ group (*p* ≤ 0.001; decrease = 31.9%) and 2,312.3 (2093.5–2725.3) U/g Hb in the POD− group (*p* ≤ 0.001; decrease = 23.7%). The decrease in BChE activity was significantly more pronounced in the POD+ group than in the POD− group (*p* = 0.002; Figure 2).

BChE activity was lower in the POD+ than in the POD− group throughout the postoperative period. Averaged across all days, the mean BChE activity was significantly lower in the POD+ group than in the POD− group (*p* < 0.001). The decrease in BChE activity was not synchronized between groups, which reached their lowest levels on different days.

The mean difference in daily mean BChE activity between groups was 187.9 [135.1–201.4] U/g Hb. After surgery, the median BChE activity was consistently below the physiological threshold (2300–7000 U/g Hb) in the POD− group from postoperative day two onwards. The BChE activity nadir in the POD+ and POD− groups occurred on postoperative day four (1745.7 [1576.6–2021.5] and 1935.8 [1726.4–2192.6] U/g Hb, respectively; *p* = 0.101).

**Figure 3:**
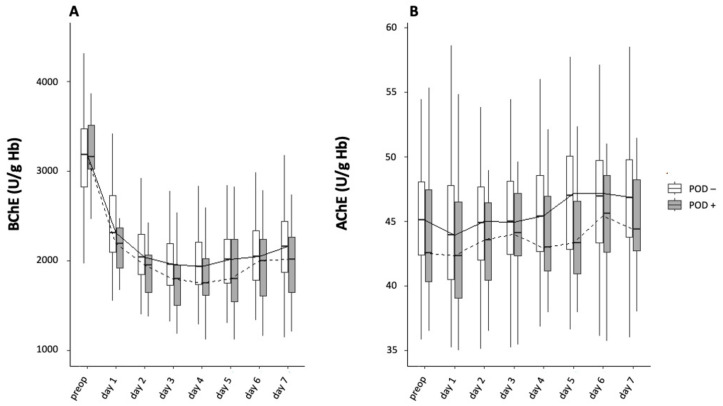
BChE (**A**) and AChE (**B**) activity preoperative and on postoperative days 1–7.

The decrease in BChE activity below preoperative activity levels persisted until the end of the observation period in the POD+ (2013.3 [1646.8–2262.9] U/g Hb) and POD− (2157.7 [1869.2–2434.3] U/g Hb) groups (Figure 3A).

In the POD+ group, BChE activity showed a mean maximum decrease of 44% below preoperative levels on postoperative day three, with a consistent decrease in BChE activity of >40% below preoperative levels until day six. In the POD− group, BChE activity reached a minimum level of 38% on postoperative day three before slightly increasing from day four onward.

AChE activity decreased to a nadir on postoperative day one in both the POD+ (42.3 [39.1–46.5] U/g Hb) and POD− (43.9 [40.3–47.9] U/g Hb) groups (*p* = 1.000), then increased on postoperative day two (43.6 [40.4–46.5] and 45.0 [42.0–47.8] U/g Hb, respectively; *p* = 1.000; Figure 3B). At the end of the observation period, AChE activity had surpassed baseline activity in both groups (44.4 [42.7–48.2] and 46.8 [43.7–49.9] U/g Hb, respectively; *p* = 1.000; Figure 3B).

**Figure 4:**
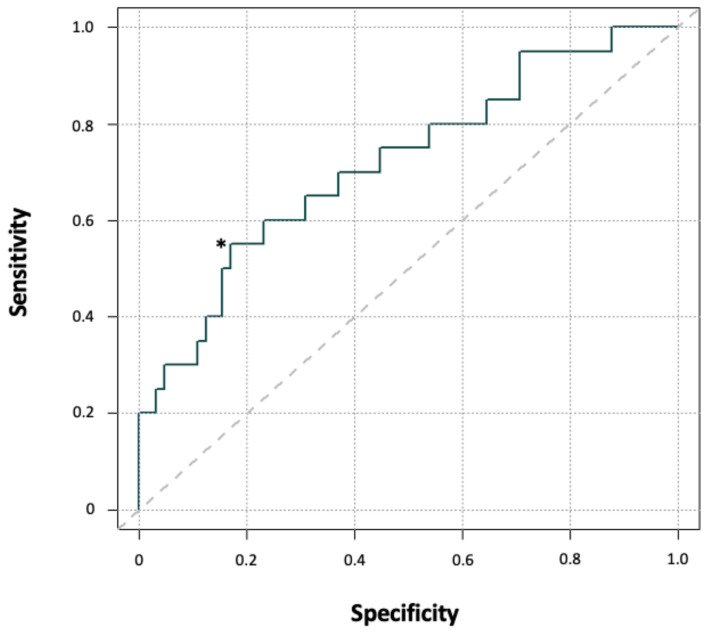
Receiver operating characteristic curve for BChE activity on postoperative day one as a predictor of POD. Key: *, optimal cut-off point that optimizes the differentiating ability when equal weight is given to sensitivity and specificity.

The decrease in BChE activity from preoperative to postoperative day one represents a potential model for predicting POD development. The area under the ROC curve (AUC), a measure of how well this parameter could distinguish between POD+ and POD− patients, was 0.72 (*p* = 0.002; Figure 4), indicating an adequate level of POD prediction.

A BChE activity decrease of ≥32% was the optimal cutoff for predicting POD (best Youden index = 1.39 with sensitivity = 0.84, specificity = 0.55). The risk of developing POD was 4.31 times higher for patients with a BChE activity decrease of ≥ 32% from preoperative baseline (*p* = 0.01). Further, the relative drop in BChE activity compared to baseline activity was associated with an increased occurrence of POD (Table 4). Analogous to BChE activity, the AUC of the AChE activity drop was calculated, which, however, was non-significant (AUC 0.54, *p* = 0.86). For this reason, an optimal threshold was not determinable.

## 4. Discussion

This study found a significant association between postoperative alterations in BChE activity and POD occurrence. The BChE activity decreased from preoperative baseline values on postoperative day one, and its degree was highly associated with POD development. The mean postoperative BChE activity throughout the study period was significantly lower in POD+ patients than in POD− patients and below physiological thresholds. However, neither preoperative nor postoperative AChE activity was associated with POD occurrence.

The overall incidence of POD in our study was 21.5%, consistent with previous reports [13,15,16,17]. Previous studies have demonstrated that changes in cholinergic activity patterns are associated with POD occurrence in different scenarios. However, implementing diagnostic biomarkers is often impractical for clinical applications since changes in biomarker patterns occur at different time points and often have no predictive value. Saha et al. [18] presented a promising approach for transferring point-of-care (POC) CHE analysis for POD into clinical practice, investigating the diagnostic value of CHE activity on POD development after mixed cardiac surgery. They identified a postoperative decrease in CHE activity of >50% as an independent risk factor for POD development, which could easily be applied to stratify patients with an increased POD risk profile. Since Saha et al. analyzed CHE activity with a laboratory assay instead of a POC measurement in a mixed cardiac surgery cohort, the results cannot be directly compared with those in our study.

This study identified a postoperative decrease in BChE activity from baseline to be strongly predictive of POD development. The most distinctive decrease occurred within the first 24 h after surgery and was significantly greater in the POD+ group than in the POD− group (31.9% vs. 23.7%, *p* = 0.002). A greater postoperative BChE activity decrease was also associated with higher odds of POD in our cohort. Therefore, POC analysis of BChE activity may be a promising marker for the early detection of cardiosurgical patients at risk for POD as a direct, easy-to-apply assay in inpatient treatment without the risk of time- or storage-related measurement errors.

Cholinergic non-neuronal-immune interactions are involved in the endogenous response to sterile and non-sterile inflammation. Changes in CHE subtype activities differ depending on the influencing factor. A rapid decrease in BChE activity was described as a physiological response to sterile inflammation after surgery and trauma, while a delayed decrease in BChE activity was described in response to sepsis [25,26,27]. In contrast, AChE activity is downregulated as part of the antimicrobial immune response and results in the control of bacterial proliferation [28,29].

Our analysis of AChE activity did not show pronounced changes during the investigation period, and AChE activity remained within reference ranges in both the POD+ and POD− groups. Simultaneously, we observed a comparable postoperative inflammatory response in the POD+ and POD− groups, measured by the leucocyte count, CRP level, and procalcitonin, and no observed sepsis during the study period. These results suggest that non-septic inflammation after CPB does not cause pronounced changes in AChE activity, and a stable postoperative course of AChE activity could be a possible biomarker for excluding acute septic encephalopathy as the cause of POD. These results are supported by Zujalovic et al., who observed decreased AChE activity in septic ICU patients, while non-septic ICU patients, even those with delirium or cognitive impairment, did not show altered AChE activity [30]. They described that the longitudinal measurement of AChE activity over several consecutive days revealed a change from baseline only in septic patients with suspected sepsis-associated encephalopathy (SAE). Therefore, AChE activity was suitable for differentiating SAE from other causes of delirium. AChE activity at the end of the observation period surpassed preoperative baseline values, consistent with the literature [13,17], contradicting a sepsis impact.

In summary, our data indicate that AChE activity is unsuitable for identifying POD in non-septic post-cardiac surgery patients. Other comparable studies reported disparate results. John et al. [13] described stable postoperative AChE activity, while Adam et al. [17] described a significant postoperative decrease associated with POD development. Unlike John et al. and Adam et al., who analyzed cholinergic activity in mixed cardiac surgery, this study analyzed only elective on-pump CABG surgery. Prolonged cross-clamp times and pre-existing variations in the cholinergic system or inflammatory status might have influenced these results.

Cerejeira et al. [15] and Adam et al. [17] described reduced preoperative cholinergic activities in patients developing POD. We observed similar preoperative cholinergic activity in the POD+ and POD− groups that were within physiological thresholds [13]. Preoperative cholinergic activity can be affected by age, malnutrition, physical capacity, inflammatory status, and individual CHE genotype [31,32]. Therefore, identifying specific reasons for preoperative reduced cholinergic activity is difficult. Due to its explorative study design, this analysis cannot offer explanations for its findings. Even though the study cohort was homogenous, conclusions should be drawn with great caution due to several confounders influencing the AChE- and/or BChE-activity, such as the etiology of the pre-existing diseases, the type of performed interventions, and the progress of the clinical conditions. Beyond, studies have so far failed to determine whether changes in the AChE- and/or BChE-activity are causative with the emergence of POD. Based on this, only assumptions can be made for the drop in BChE with simultaneously stable AChE activities.

For a long time, the physiological role of BChE remained unclear, leading to its declaration as an “orphan enzyme”. This assumption was drawn because of the knowledge that genetic mutations eliminating BChE activity do not result in a cognitive dysfunction [33]. In summary, BChE was assumed not to play a relevant role in human physiology; however, recent studies changed this assumption. Besides metabolizing exogenous bioactive esters in the diet or in medicines, BChE regulates ghrelin activity, which is important for the stress response, neural circuitry, substantial inactivation, and unusually high levels of internalization [34,35]. Ghrelin is also involved in the regulation of the psychosocial status, memory, and learning [36].

The affection and modulation of BChE activity and therefore a potential dysregulation of the stress response and neuronal activity might be relevant in the development of POD. On the other hand, until today, the role of AChE in this context is still unknown.

Falling BChE activities with primary stable AChE activities were described in various settings. For example, John et al. analyzed cholinergic activity levels after cardiac surgery with POD. They showed that AChE increased and BChE decreased within the first 3 days after surgery. The authors supposed that the perioperative change in AChE and BChE activity might possibly be explained by an interaction of AChE and BChE and the use of a CPB [13]. Contrary, Michels et al. [37] presented data showing that BChE activity decreased also significantly after transapical aortic valve implantation, an operative procedure without the use of CPB. This finding contradicts the hypothesis that the release of micro-RNAs during CPB is modulating cholinergic homeostasis. Independently of cardiac surgery, micro-RNAs are known to increase AChE activity with a subsequent deterioration in BChE activity [38]. Another explanation might be the way in which AChE and BChE are transported in the blood. While AChE is bound to erythrocytes, BChE is produced in the liver and released into the plasma where the enzyme is dissolved. Since CPB influences both erythrocytes and the plasma volume, it might influence the concentration of both enzymes. Last, the discrepancies in the mentioned studies might also be attributed to different assays for measuring enzyme activities and considerable differences in the study design. In conclusion, our results indicate that intraoperative variables during cardiac surgery affect cholinergic homeostasis rather than preoperative factors.

In our study, POD+ patients were older, had higher EuroSCOREs, and had higher incidences of type II diabetes mellitus and a trend toward COPD. In addition, the POD+ group had prolonged periods of CPB and aortic cross-clamping. Therefore, regional cerebral hypoperfusion and inflammatory activation may have been more common in this group, as described previously [7,10].

The generation of cerebral microembolism during and after CPB might be associated with regional cerebral hypoperfusion [7,39,40], which can derive from gas bubbles, biological aggregates (i.e., coagulated thrombus), or inorganic debris (i.e., disrupted calcified plaques) [41]. The “wash-in” of these small fragments can result in a silent ischemia not resulting in a clinical stroke but in subclinical damage. POD has been discussed as one of the potential consequences of microembolism; however, currently, the scientific community remains undecided on its influence on cognitive impairment after cardiac surgery because most studies were too underpowered to prove a relationship [42,43,44].

Moreover, the POD+ group had a lower hemoglobin level (anemia) before and during surgery and a higher volume of transfused RBCs. These factors have previously been described as POD risk factors in cardiosurgical patients [13,17,45,46], supporting the validity of the overall data set. Intraoperative blood loss might be a surrogate for surgical complexity, duration, and/or complications that may increase POD risk [47]. The POD+ group had longer ventilation durations and ICU stay lengths, as described in previous studies [2,48]. Unlike other studies [2,13,49,50], we observed no difference in hospital stay length.

Our study had some limitations. It was conducted in a single center and comprised only cardiosurgical patients. CHE deficiency syndromes represent a potential confounder in studies of perioperative cholinergic disturbance. However, their effect is unclear. In addition, while polymorphisms and mutations in AChE and BChE are common in the general population [51], their impact on BChE and AChE activities after cardiac surgery is unknown. Variations in CHE genotypes in Alzheimer’s disease have been associated with differences in disease phenotype [32]. Future studies should consider analyzing these potential confounders. Last, sample size calculation was not feasible because the study displayed a sub-study of a prospective study with different primary study parameters. This study’s strengths include examining preoperative baseline CHE activities and using patient questionnaires to determine POD. Moreover, activities were measured at a consistent time every day for seven days after surgery to analyze the temporal development of POD.

We did not analyze the anticholinergic effect of perioperative medications as presented in the Anticholinergic Cognitive Burden Scale [52]. Intraoperative drug administration was standardized and not adapted for this study. It has been previously verified that the applied pharmacological agents do not inhibit cholinergic activity at therapeutic serum concentrations [53,54]. Furthermore, we considered the medication regime in the ICU as not adjustable. Certain medications, even those with an elevated anticholinergic burden, cannot be replaced because alternatives do not exist. Medical therapy was used to optimize individual clinical status.

The prevention of POD, even with the possibility of the early identification of patients with an increased risk profile, represents a complex and challenging task.

According to current international guidelines on the management of POD, the role of the prevention of POD should be emphasized in daily clinical practice.

The hemostasis of cholinesterase plays a significant role in the development of POD, which is also supported by this study. Therefore, its prevention should be addressed carefully. First, anticholinergic medication should be avoided. This includes anesthesia-related drugs (i.e., atropine) but also the patients´ daily medications. Particularly, patients receiving a polypharmaceutic regime should be managed with caution regarding their anticholinergic load [54,55]. Second, the benefit of cholinesterase inhibitors can be discussed. Even though this study showed a potential relationship between BChE and the development of POD, it was not able to support the use of cholinesterase inhibitors, which have been described as beneficial by some studies. Contrary, more recent studies, including a meta-analysis, were not able to show a therapeutic effect and one study even resulted in a harming effect induced by rivastigmin [56,57].

Preventive strategies also utilize non-pharmacologic multicomponent initiatives.

One of the most non-pharmacologic consistently effective delirium prevention strategies involves cognitive orientation, social support, sleep protocol implementation, assistance with nutrition and mobilization, and education for health-care staff, and is found in the Hospital Elder Life Program (HELP) [58], significantly reducing delirium incidence, risk of falls, and health-care costs [59].

Further studies should address the interaction of CHE subtypes in septic and sterile inflammation and variations in CHE genotypes. In addition, they should investigate potential alterations in cholinergic receptors responsive to perioperative stress. Future large-scale studies are warranted.

## 5. Conclusions

After CABG surgery, POD development was associated with an early decrease in BChE but not AChE activity. Intraoperative rather than preoperative factors appear to be the primary interfering factors in cholinergic homeostasis. An early pre- to postoperative BChE reduction analysis may represent a practicable tool in clinical POD risk stratification. Our results support previous findings and foster the current concept of cholinergic disruption and the cholinergic deficit hypothesis in POD development.

## Figures and Tables

**Table 1:** Group-wise baseline patient characteristics.

Characteristic	POD+ (*n* = 20)	POD− (*n* = 73)	*p*-Value
Age (years)	71.5 (63.8–76.3)	64 (56–71)	0.003
Female sex	3 (15.0)	9 (12.3)	0.717
Body mass index (kg/m²)	28.9 (25.7–32.4)	29.4 (27–32.1)	0.581
Atrial fibrillation	3 (15.0)	19 (26.0)	0.385
COPD	5 (25.0)	6 (8.2)	0.054
DM II	13 (65.0)	22 (30.1)	0.008
EuroSCORE	1.4 (1.2–1.8)	0.9 (0.7–1.2)	<0.001
ASA–PSCS			
II	0	7 (9.6)	-
III	19 (95.0)	57 (78.1)	-
IV	1 (5.0)	9 (12.3)	-

Values are presented as medians with interquartile ranges or numbers with percentages. Key: POD, postoperative delirium; COPD, chronic obstructive pulmonary disease; DM II, diabetes mellitus type II; ASA–PSCS, American Society of Anesthesiologists Physical Status Classification System, roman numerals represent a graduation for the risk classification of surgical patients (increasing from I to IV).

**Table 2:** Group-wise perioperative laboratory findings.

Characteristic	POD+	POD−	*p*-Values
**Preoperative**			
ALT (U/L)	21 (17.0–32.5)	25.5 (17.75–43.0)	0.289
GGT (U/L)	26.5 (19.0–39.5)	34.0 (25.0–48.25)	0.161
HBA1c (%)	6.5 (6.0–7.0)	5.8 (5.5–6.4)	0.003
Glucose (mg/dL)	164 (154.0–199.0)	102.5 (92.3–126.5)	0.039
**Intraoperative**			
Hb (g/dL)	10.0 (9.3–11.2)	11.1 (10.0 –12.2)	0.016
Thrombocytes (×10^6^/L)	157.5 (106.0–167.0)	169 (140.25 –205.5)	0.042
GGT (U/L)	13 (10.8–21.3)	12 (16.5–34.0)	0.018
**Postoperative**			
Hb (g/dL)	97.5 (89.5–104.5)	109 (97.0–117.0)	0.002
Erythrocytes (×10^6^/µL)	3.3 (2.9–3.5)	3.6 (3.2–3.9)	0.009
Hematocrit (%)	0.30 (0.26–0.32)	0.32 (0.28–0.34)	0.016
MCHC (g/dL)	336.5 (328.8–339.3)	341 (334.0–347.0)	0.002
Thrombocytes (×10^6^/L)	173.5 (121.8–196.0)	187.0 (160.0–220.0)	0.015
GGT (U/L)	20 (12.0–28.0)	24.0 (20.5–33.5)	0.076
ALP (U/L)	41 (35.0–44.0)	54.5 (43.5–63.8)	0.009

Values are presented as medians with interquartile ranges or numbers with percentages. Key: POD, postoperative delirium; ALT, alanine aminotransferase; GGT, gamma-glutamyl transpeptidase; HBA1c, human glycated hemoglobin; Hb, hemoglobin; MCHC, mean corpuscular hemoglobin; ALP, alkaline phosphatase; RBC, red blood cell; CPB, cardiopulmonary bypass; LOS-ICU, length of stay in the intensive care unit; LOS-Hospital, length of stay in the hospital.

**Table 3:** Group-wise surgical and anesthetic characteristics.

Characteristic	POD+ (*n* = 20)	POD− (*n* = 73)	*p*-Values
**Surgical and anesthetic characteristics**			
CPB-time (min)	107.0 (93.3–117.0)	77.0 (66.8–90.0)	<0.001
Cross-clamp time (min)	71.0 (55.5–80.3)	53.0 (45.0–67.0)	0.011
Anesthesia duration (min)	298.5 (261.0–327.0)	262.0 (245.0–303.0)	0.024
Surgery duration (min)	223.0 (195.8–238.9)	192.5 (164.0–225.0)	0.046
RBC transfusion during observation period (mL)	450.0 (0.0–600.0)	0.0 (0.0–0.0)	<0.001
Ventilation duration (h)	20.8 (17.0–24.2)	12.3 (9.4–16.6)	<0.001
LOS-ICU (d)	2.9 (5.2–7.2)	1.1 (0.9–2.0)	<0.001
LOS-Hospital (d)	11.0 (9.3–13.8)	10.0 (8.0–12.0)	0.125
In-hospital mortality	0	0	-

Values are presented as medians with interquartile ranges or numbers with percentages. Key: CPB, cardiopulmonary bypass; ICU, intensive care unit; LOS, length of stay; POD, postoperative delirium; RBC, red blood cell concentrate.

**Table 4:** Odd’s ratios for the development of POD dependent on the relative drop of butylcholinesterase.

Timepoint	Drop to Baseline (%)	Odd´s Ratio	*p*-Values
Day 1	20	3.89 (0.81–37.62)	0.08
Day 1	25	3.62 (1.10–14.22	0.02
Day 1	30	3.88 (1.24–12.86)	0.01
Day 1	35	4.66 (1.18–18.77)	0.01
Day 1	40	16.27 (1.48–844.38)	0.01
Day 1	45	N.A.	0.01
Day 2	20	N.A.	0.57
Day 2	25	N.A.	0.03
Day 2	30	2.52 (0.63–14.80)	0.26
Day 2	35	1.90 (0.62–6.32)	0.31
Day 2	40	5.47 (1.71–18.63)	0.002
Day 2	45	5.51 (1.22–26.45)	0.01
Day 3	20	N.A.	1.0
Day 3	25	N.A.	0.33
Day 3	30	2.78 (0.34–129.17)	0.45
Day 3	35	5.90 (1.25–13.11)	0.01
Day 3	40	3.66 (1.15–13.11)	0.02
Day 3	45	3.58 (0.95–13.28)	0.04

Key: N.A., not applicable.

## Data Availability

The datasets used in this study are available from the corresponding author upon reasonable request.
